# Political and social analysis of the impact of national identity and gender roles on national stability (in the context of Kazakhstan)

**DOI:** 10.3389/fsoc.2026.1853594

**Published:** 2026-06-30

**Authors:** Nursulu Nurpeissova, Zhengisbek Tolen, Serikkhan Zhuzeyev, Makhpal Syzdykova, Aigul Abdiramanova

**Affiliations:** 1 Al-Farabi Kazakh National University, Almaty, Kazakhstan; 2 Turan University, Almaty, Kazakhstan; 3 Korkyt Ata Kyzylorda University, Kyzylorda, Kazakhstan

**Keywords:** gender roles, mixed research method, national identity, society, state stability

## Abstract

**Introduction:**

In post-Soviet societies, the transformation of national identity and gender roles has become a pressing issue for state stability. However, there is little empirical research on the combined impact of these factors on social cohesion. This study aims to analyze the relationship between attitudes toward national identity, gender equality, and national stability in Kazakhstan.

**Methods:**

The research design used a mixed-methods approach, combining quantitative and qualitative methods. In the first phase, 284 participants were surveyed using purposive sampling targeting urban youth and university networks. The data were analyzed using multiple linear regression and factor analysis. In the second phase, three focus group interviews were conducted to provide a deeper understanding of the statistical findings.

**Results:**

Empirical results show that the variable “uniqueness and gender integration” is the strongest predictor of national stability (β = 0.254, *p* < 0.001). National identity itself also has a positive effect on stability (β = 0.220, *p* < 0.001). However, the attitude to gender roles in isolation did not show a direct statistically significant effect on national stability (beta = −0.075, *p* = 0.168). Qualitative analysis explains this phenomenon through society's rejection of pure egalitarian modernization as a guarantee of state security, and through latent resistance to foreign gender discourses isolated from the national context.

**Discussion:**

The study shows that political stability in Kazakhstan is not achieved through simply imposing gender equality, but rather through integrating it as a natural part of the national historical code. This article proposes a new approach to implementing ideological policies and gender reforms, focusing on national security and social wellbeing through a communication paradigm that integrates gender equality into the national narrative.

## 1 Introduction

For independent states in the post-Soviet region, including Kazakhstan, nation-building and national identity formation are crucial to ensuring state stability. After gaining independence, Kazakhstan has faced a challenging path typical of societies in transition, seeking a balance between civic and ethnic-national identities. Although the role of national identity in shaping institutional trust and social harmony has been widely studied in political science and sociology, these processes continue to evolve amid new global challenges.

In the current scientific literature, studies on national stability are often limited to the discourse of political elites, language policy, and interethnic relations. However, the intersection of gender roles and ethno-national factors is often overlooked, as is the convergence of national values in Kazakhstan with the egalitarian views brought about by globalization, which are creating new ideological conflicts in society. The state relies on traditional family values to preserve the ethno-national code, while democratic modernization and global information openness call for a rethinking of the roles of women and men in society. Measuring the complex impact of these opposing processes on state stability empirically is a major research gap in today's science.

To address this gap, this study aims to answer the key question: how do the strengthening of national identity and the conflict between traditional and modern gender roles in Kazakhstan influence national stability?

In order to answer the research question and conduct an empirical analysis based on the constructivist paradigm, we propose the following hypotheses:

1. A high level of civic national identity contributes positively to increased institutional trust in state institutions and serves as a direct determinant of political stability.

2. Despite the endorsement of traditional gender roles and neo-traditionalist political discourse, gender positions do not directly predict national stability when considered as an isolated variable.

3. The synergistic integration of modernization values and egalitarian gender attitudes, together with the construct of national identity, has the strongest positive effect on long-term political stability based on democratic principles.

This study aims to re-examine national stability, not only within a political and institutional framework, but also in its complex intertwining with gender and ethnic-cultural factors. The empirical findings will provide a scientific basis for enhancing ideological policies in transitional societies and for developing new, hybrid models of social cohesion.

### 1.1 Literature review

In contemporary political science, socio-political stability is directly linked to the process of constructing a national identity. This literature review analyses modern scientific discourse, starting with classical theories of nation-building and incorporating ethno-cultural, global, and gender aspects of identity.

To understand the relationship between national identity and state stability, we need to rely on the constructivist paradigm proposed by Anderson (1983), Hobsbawm (2012), and Gellner (1983). Anderson (1983), a representative of classical constructivism, describes the nation as an “imagined community” grounded in deep social integration. Gellner (1983) sees the nation as a product of industrialization and considers the coincidence of ethnic and political borders a key condition. Hobsbawm (2012) continues this line of thought, arguing that national myths are artificially created in response to socio-economic needs.

However, unlike these theories based on Western experiences, the formation of national identity in post-Soviet states like Kazakhstan is not solely determined by economic factors. Instead, state policies aimed at reviving historical memory and promoting inter-ethnic harmony play a significant role in shaping national identity.

As an alternative to pure constructivism, Smith (2009) explains the formation of national identity through an ethno-symbolic approach, which includes cultural heritage and myths. While Ignatieff (1993) warns of the dangers of ethnic exclusivity for state stability, Brubaker (1996), 2004) proposes the concept of “nationalizing states” and suggests considering ethnicity as a cognitive category rather than a strictly defined physical unit. The main focus of scientific discussion in this area is the dichotomy between civic and ethnic nationalism. However, contemporary research strongly challenges this traditional division. For instance, while Kuzio (2002) regards the purely civic nation as a “myth,” Brubaker (1999) dismisses this dichotomy as a “Manichean myth,” arguing that in reality, both forms of nationalism are closely interconnected.

Shulman (2002) therefore argues for reconsidering the traditional dichotomies of civic/ethnic and Western/Eastern in the study of nationalism. This theoretical conclusion is supported by empirical evidence from societies in transition, such as Poland. Jamroz-Dolinska et al. (2023) identify the transition of society toward inclusive civic nationalism. Piwoni and Mußotter (2022) view this process as a continuum between nationalism and democratic patriotism, arguing that civic identity strengthens stability by incorporating ethno-cultural elements.

In this context, the stability of Kazakhstan's society directly depends on the balance between civic identity and ethnic diversity. Dave (2007) explains this process in terms of ethnicity, language, and power relations. Beachain and Kevlihan (2013), on the other hand, view it as a delicate balance between the state's support for both civic and ethno-nationalistic directions. Burkhanov (2017), meanwhile, analyses the interplay between Soviet heritage and current politics in shaping identity. Laruelle (2015), finally, systematizes the ideological search through three discursive paradigms: “being Kazakh,” “Kazakhstanness,” and transnationalism.

Empirical studies have revealed the specific nature of identity construction processes. Sharipova et al. (2017) examined the determinants of civic and ethnic nationalism in Kazakhstan, while Rees and Williams (2017) analyzed Kazakhstani identity through the lens of supra-ethnic identity and citizenship. The spatial territorialization of the sense of national belonging (Rees et al., 2021) and the rhetorical transformation demonstrate the complexity of forming a new Kazakhstani nation. This multilevel nature of identity is closely linked to the modernization process of state ideology and public consciousness (Bashmakov et al., 2020).

The multi-layered nature of identity is supported by recent research. For instance, Sharipova et al. (2017) argue that “Kazakhstani identity” is not homogeneous and that the coexistence of civic and ethnic elements within it is the foundation for interethnic harmony and stability. These layers of identity are often reinforced through the educational system (Sarmini et al., 2023) and cultural transmission through children's literature (Seisenbiyeva et al., 2024). Balmagambetova et al. (2020) emphasize the significance of linguistic and cultural symbols in collective memory, using the concept of “national precedents.”

Contemporary research emphasizes the impact of digitalisation and globalization on national identity. Humayun (2023) argues for the role of sports journalism in this process, while Capurro et al. (2024) highlight the importance of digital cultural heritage infrastructure in constructing civic identity.

Globalization poses challenges for the preservation of cultural identity, as noted by Mazurkevych et al. (2024). However, its integration with the national dimension can lead to the emergence of new hybrid models, as Li et al. (2025) argue. In such a complex environment, social capital (Christensen, 2024) and multicultural policies (Baycar, 2023) are essential for societal stability. However, Ibrayeva and Zhanbulatova (2022) caution against purely institutional approaches to addressing ethno-linguistic diversity and social inequality, as these can hinder progress in this area.

National identity and political stability are closely linked to ideological and cultural processes. Schiek (2022) examines the depoliticisation of civic participation in Kazakhstan through “consultative ideology,” while Manukyan (2025) considers the modernization of the post-Soviet identity as a major historical transformation. These efforts are particularly evident in the cultural sphere. The intentional “ambivalence” of national symbols enhances political legitimacy, as demonstrated by Insebayeva and Insebayeva (2022). Meanwhile, the capital's sculptures subtly express nationalism in the urban landscape, as Fauve (2015) notes.

The nature of these ideological tools can be explored through the archaeology of post-Soviet regimes (Kudaibergenova, 2017) and through the evolution of cinema (Isaacs, 2018). In general, culture and artistic expression serve as essential tools for social cohesion (Kuppili, 2022), strengthening the concept of “national sovereignty” (Ospanova et al., 2024).

These ideological constructs are directly challenged at the level of linguistic and interethnic relations in society. Fierman (1998) examines language and identity in political documents, while Faranda and Nolle (2011) analyse ethnic boundaries between the titular nation and the Russian diaspora in Kazakhstan and Kyrgyzstan. In this process, Peyrouse (2008) sees Russians as an “imperial minority,” while Werner et al. (2017) identify the policy of “exclusion of privilege” in the migration of ethnic repatriates.

The full picture of identity is enriched by postcolonial and social constructivist perspectives. Kudaibergenova (2016) critically analyses the use and distortion of postcolonial discourses, while (Heathershaw (2016) uses this perspective to assess the relationship between external stability and internal vulnerability in Central Asia. Contemporary research grounds national identity as a spatial-symbolic process, constructed through territorial discourse and the opposition between “us” and “others” (Paasi, 2022). Territory is considered an important symbolic element in this process (Kadyrzhanov, 2023). Post-Soviet identity is still being formed at the intersection of historical heritage and new political discourses, influenced by Soviet narratives (Khalid, 2021; Tergembay et al., 2025).

Modern research emphasizes the influence of globalization and new media on the formation of national identity. Li et al. (2025), for example, argue that national and global identities are not mutually exclusive but rather hybrid, which is important for multicultural societies like Kazakhstan.

In the context of globalization, the preservation of cultural identity is ensured through the continuity of youth traditions (Gabitov et al., 2025) and cultural communication in education (Kassymova, 2023). Additionally, the information space plays a crucial role, as the unique features of youth media consumption, particularly in border regions, shape new dimensions of the nation-building process (Petrov et al., 2025).

In addition, the transformation of national identity and public consciousness is clearly reflected through the lens of tradition, religion, and gender. Bolysbayeva et al. (2024), for example, explain the gender aspect of radical processes that threaten social stability through the phenomenon of the feminization of extremism. Syzdykova et al. (2024) analyse the transformation of Kazakh traditions in the context of globalization, using the southern regions of Kazakhstan as a case study. Kudaibergenov et al. (2025) provide a comprehensive study of the connection between these traditional values and modern social dynamics, while Abikenov et al. (2025) substantiate the role of religious spirituality in shaping public consciousness and regulating interpersonal relations in the southern regions of Kazakhstan. All these factors demonstrate that the intertwining of national identity and gender roles is essential for social stability.

Furthermore, this broader socio-cultural transformation is deeply rooted in the cultural framing of national values (Abikenov et al., 2019) and the continuous adaptation of the educational system to modern labor market demands and ethnopedagogical needs (Syzdykova et al., 2022; Zhuzeyev and Zhailauova, 2025). At the same time, the rapid digitalization of the economy and media space significantly influences societal values, actively transforming traditional gender stereotypes (Baskynbayeva et al., 2024) and introducing new behavioral patterns through globalized digital platforms (Fauzi et al., 2026).

In political science and sociology, the study of national identity and gender has often been conducted in isolation from one another. However, there is a significant gap in the literature regarding the integration of these two concepts as a predictor of state stability. Empirical studies examining the combined effect of national identity and gender on state stability are scarce.

As intersectional analysis reveals, in transition societies, the national ideology based on neo-traditionalism and the egalitarian values generated by modernization can often lead to conflict. Therefore, socio-political stability does not depend solely on national identity or gender equality, but rather on their combination. Only when a society can organically integrate national cultural codes with modern egalitarian gender values can it achieve high levels of institutional trust and internal stability.

For the purposes of this study, the term “national historical code” refers to the collective system of historical memory, cultural traditions, language, symbolic heritage, and shared values that ensure the continuity of national identity across generations. In Kazakhstan, this code functions as a key mechanism for preserving social cohesion while adapting to contemporary social and political transformations.

### 1.2 Materials and methods

In this study, a mixed-method research design was employed to provide comprehensive answers to the research questions. The research consisted of two stages: the first involved a quantitative survey to test hypotheses, and the second a qualitative focus group to deepen understanding of the survey findings.

Theoretical and political-social analyses were based on neo-institutionalist and intersectional approaches. The goal was to combine quantitative and qualitative methods to provide a more complete understanding of the topic.

A total of 284 respondents participated in the quantitative part of the study. Data collection was conducted using a purposive sampling method. Participants were intentionally recruited through university networks and youth-oriented social media channels. This specific demographic was targeted because young, urban populations are at the forefront of the intersection between global modernization values and traditional national codes, making them the most relevant group for studying evolving gender and civic identity discourses. The demographic structure of the respondents was as follows: by gender, 60.6% were women and 39.4% were men; by age, young people aged 18–24 (34.5%) and 25–34 (36.6%) predominated. 47.2% of the participants had higher education, and by place of residence, 69.0% were urban, 31.0% were rural. In terms of ethnic composition, the share of Kazakhs was 69.7%, in line with the overall demographic composition of Kazakhstan.

A specially designed questionnaire consisting of 30 questions was used to collect empirical data. All questions were measured on a 5-point Likert scale (1—“strongly disagree”, 5—“strongly agree”).

The questionnaire used in the study was divided into four main constructs. The first indicator, “National Identity” (NI_mean), consisted of eight questions aimed at measuring respondents' sense of civic belonging and appreciation of common values (e.g., the statement: “Common values characteristic of Kazakhstan unite citizens”). The second construct, “Attitudes to Gender Equality” (GR_mean), included eight questions that determined positions on traditional and modern gender roles in society. The third measure was the variable “National Stability” (NS_mean), consisting of eight questions that assessed public agreement and political interethnic harmony. The final, fourth construct—“Uniqueness and Gender Integration” (NG_mean) combined six questions designed to analyze the compatibility of national tradition and gender equality. In this study, “uniqueness” refers to the preservation of Kazakhstan's distinctive historical, cultural, linguistic, and symbolic identity within the process of social modernization. It reflects the capacity of society to maintain cultural continuity while simultaneously adapting to democratic and egalitarian values.

To control for the authenticity and reliability of the responses, six reverse items (REV) were included in the questionnaire and recoded before statistical analysis.

Focus group discussions were held to explain statistical deviations in the quantitative analysis results. The study involved three focus groups (8–10 participants each) with different social statuses (students, working youth, and representatives of the older generation). The moderator collected respondents' latent opinions on the relationship between national stability and gender roles through semi-structured questions.

The moderator guide included core questions such as: “How do you perceive the balance between national traditions and modern gender roles?”, “Does gender equality directly impact the stability of our country?” and “How do Western gender discourses fit into our national context?” The discussions were conducted primarily in the Kazakh language, with bilingual code-switching accommodated to ensure participants' comfort. All audio recordings were fully transcribed and translated into English for analytical purposes. Acknowledging their positionality, the research team comprising Kazakhstani scholars carefully managed the discussion environment to ensure that their own academic backgrounds and gender identities did not unduly influence participants' open expressions. Qualitative data were processed using a rigorous thematic analysis approach, where transcripts were inductively coded to identify recurring patterns. The quotes included in the results were specifically selected because they are highly representative and reached thematic saturation across the distinct groups.

Quantitative data were processed using statistical software. Descriptive statistics were performed to check for missing values and identify general trends. Linear relationships between variables were tested using Pearson correlation analysis. Multiple linear regression was used to predict the main factors affecting national stability (NS_mean), and model validity was assessed using analysis of variance (ANOVA). Factor analysis, including a Scree plot and parallel analysis, was conducted to assess the structural integrity and factor loadings of the study variables. Qualitative focus group data were processed using thematic analysis.

## 2 Results

This section presents the results of a quantitative empirical analysis conducted to test the research hypotheses. First, descriptive statistics, Pearson correlation, and multiple linear regression are used to identify the relationships between key variables and predictors of national stability. Then, the structural integrity of the obtained quantitative patterns is assessed through factor analysis, and the statistical validity of the proposed research model is confirmed. As shown in Table 1, the socio-demographic characteristics of the respondents indicate diverse representation across gender, age, education, and ethnicity.

**Table 1:** Socio-demographic characteristics of respondents.

Variable	Category	*n*	%
Gender	Male	112	39.4
	Female	172	60.6
Age	18–24	98	34.5
	25–34	104	36.6
	35–44	52	18.3
	45+	30	10.6
Education	Secondary	34	12.0
	Special secondary	76	26.8
	Higher	134	47.2
	Post-secondary	40	14.1
Place of residence	City	196	69.0
	Village	88	31.0
Ethnic composition	Kazakh	198	69.7
	Russian	46	16.2
	Other	40	14.1
Language	Kazakh	172	60.6
	Russian	88	31.0
	Bilingual	24	8.4
Social status	Student	96	33.8
	Employed	148	52.1
	Unemployed	40	14.1

Source: Compiled by the authors based on an online survey conducted in 2026.

A total of 284 participants took part in the study. Most participants were women (60.6%), with men accounting for 39.4% of the total. Regarding age, the majority of respondents were aged between 25 and 34 (36.6%), followed by those aged 18 to 24 (34.5%). This indicates that young people were mainly represented in the study.

Regarding education, most participants had higher levels of education (47.2%), thereby enhancing the quality of the collected data. As for place of residence, most respondents (69%) lived in urban areas, while 31% lived in rural areas. Ethnically, Kazakhs made up the majority (69.7%), reflecting the country's demographic makeup. Overall, the socio-demographic composition of the respondents encompassed various groups, aligning with the study's objectives and enabling generalization of the results. The descriptive statistics and correlations among the study variables are presented in Table 2.

**Table 2:** Detailed description of the survey scales.

Variable name	Scale name	Questions (code)	Question number	Brief description (question texts)
NI_mean	National identity	NI1–NI8	8	NI1—I feel like a full member of Kazakhstani society;
				NI2—I believe that the common values inherent in Kazakhstan unite citizens
				NI3—The history and cultural heritage of Kazakhstan are important to me
				NI4—State symbols are of particular importance to me
				NI5—It is important for different ethnic groups in Kazakhstan to have a common civic identity
				NI6—A common national identity strengthens social harmony in the country
				NI7—I believe that there is an idea of a common future that unites the citizens of Kazakhstan
				NI8—National identity is an important factor for the stability of a country
GR_mean	Approach to gender equality	GR1–GR8	8	GR1—The traditional roles of men and women in society should be preserved
				GR2—A man should be the main breadwinner in the family
				GR3—For a woman, family and raising children are more important than a career
				GR4—Men and women should participate equally in public and political life
				GR5—The presence of women in leadership positions has a positive impact on the development of society
				GR6—Gender equality strengthens justice in society
				GR7—It is normal for girls and boys to have different social demands in society
				GR8—Gender roles in modern Kazakhstan should be flexible and changeable
NS_mean	National stability	NS1–NS8	8	NS1—Social harmony in Kazakhstan is a fundamental condition for the country's stability
				NS2—Citizens' trust in each other strengthens national stability
				NS3—Interethnic harmony directly affects the country's stability
				NS4—A society with social justice is stable
				NS5—Gender equality has a positive impact on national stability
				NS6—The weakening of common national values threatens the stability of society
				NS7—Mutual respect and tolerance in society strengthen political and social stability
				NS8—Citizens' sense of shared responsibility for the fate of the country increases national stability
NG_mean	Identity and gender integration	NG1–NG6	6	NG1—National identity should be compatible with the principles of gender equality
				NG2—Gender justice does not weaken national unity, but rather strengthens it
				NG3—Gender equality can be developed while preserving traditional values
				NG4—Gender inequality can exacerbate internal conflicts in society
				NG5—National identity should be associated with providing equal opportunities for all citizens
				NG6—Equality of men and women in public life contributes to the stability of the country
REV	Reverse questions	REV1–REV6	6	REV1—National identity is not very important for modern Kazakhstan
				REV2—The stability of society does not depend on the common values of citizens
				REV3—Gender equality has no effect on the stability of the country
				REV4—Equal rights for men and women contradict national traditions
				REV5—Kazakh society is united not by a common national identity, but only by personal interests
				REV6—The issue of gender roles is not related to national stability

Source: Compiled by the authors based on an online survey conducted in 2026.

All questions were rated on a five-point Likert scale (1—“strongly disagree,” 5—“strongly agree”).

Each scale consists of several statements, and their averages were calculated to form the corresponding composite variables (NI_mean, GR_mean, NS_mean, and NG_mean).

Reverse statements (REV) were included to check the reliability of the responses and were recoded before analysis.

The descriptive statistics for the study variables are presented in Table 3. A total of 284 valid responses were collected for all variables, and no missing data were recorded.

**Table 3:** Descriptive statistics for study variables.

Descriptive statistics				
Variable	NI_mean	GR_mean	NS_mean	NG_mean
Valid	284	284	284	284
Missing	0	0	0	0
Mean	4.004	3.015	3.164	3.202
Std. deviation	0.424	0.198	0.439	0.467
Minimum	2.500	2.500	2.125	2.000
Maximum	5.000	3.625	4.625	4.333

Source: Compiled by the authors based on an online survey conducted in 2026.

The indicator of national identity was high (mean = 4.004, standard deviation = 0.424), indicating that respondents have a strong sense of belonging to Kazakh society and appreciate national values.

Attitudes toward gender equality were found to be at a medium level (mean = 3.015, standard deviation = 0.198), suggesting a mix of traditional and modern views on gender roles.

Perceptions of national stability also showed a moderate level (mean = 3.164, standard deviation = 0.439), indicating that participants viewed societal stability as moderate.

The indicator of uniqueness and gender integration was at a moderate level (*M* = 3.202, SD = 0.467), indicating that the harmony between national values and gender equality is in the process of formation. The minimum and maximum values of the variables lie within the scale's range, indicating that the data are close to a normal distribution.

Pearson correlation analysis was conducted to determine the relationships between the research variables (Table 4).

**Table 4:** Pearson correlation coefficients between study variables.

Pearson's correlations					
Variable	Estimate	NI_mean	GR_mean	NS_mean	NG_mean
1. NI_mean	Pearson's *r*	–			
	*p*-value	–			
2. GR_mean	Pearson's *r*	−0.034	–		
	*p*-value	0.564	–		
3. NS_mean	Pearson's *r*	0.359	−0.092	–	
	*p*-value	<0.001	0.124	–	
4. NG_mean	Pearson's *r*	0.536	−0.036	0.375	–
	*p*-value	<0.001	0.543	<0.001	–

Source: Compiled by the authors based on an online survey conducted in 2026.

The results showed a statistically significant positive relationship between national identity and national stability (*r* = 0.359, *p* < 0.001). This means that as national identity increases, respondents value national stability more.

In addition, a strong positive relationship was found between national identity and gender integration (*r* = 0.536, *p* < 0.001). This indicates that national identity contributes to the formation of a concept of harmony that includes gender equality.

A significant positive relationship was also observed between identity and gender integration and national stability (*r* = 0.375, *p* < 0.001), indicating that the higher the level of integration, the greater the perception of societal stability.

The relationship between gender roles and national stability is not statistically significant (*r* = −0.092, *p* = 0.124). Similarly, the relationships between gender roles and national identity (*r* = −0.034, *p* = 0.564) and integration (*r* = −0.036, *p* = 0.543) were also not significant.

These results indicate that gender role attitudes are not a key factor in the model under study, whereas national identity and integration are.

To identify the factors affecting national stability, we conducted a multiple regression analysis (Table 5). The results showed that the model with the independent variables had a significant increase in explanatory power compared to the original model.

**Table 5:** Description of the regression model for predicting national stability.

Model summary—NS_mean				
Model	*R*	*R* ^2^	Adjusted *R*^2^	RMSE
M_0_	0.000	0.000	0.000	0.439
M_1_	0.426	0.181	0.173	0.399

M_1_ includes NI_mean, GR_mean, NG_mean.

Source: Compiled by the authors based on an online survey conducted in 2026.

In particular, the correlation coefficient for the *M*1 model was *R* = 0.426 and the coefficient of determination, *R*^2^, was 0.181, indicating that the variables of national identity, gender roles, and identity and gender integration explained 18.1% of the variation in national stability. The adjusted coefficient of determination (Adjusted *R*^2^) was 0.173, indicating the model's stability and reliability. Additionally, the reduction in root mean square error (RMSE) from 0.439 to 0.399 suggests improved predictive accuracy. Overall, these results suggest that the studied variables are important in explaining national stability.

An analysis of variance (ANOVA) was performed to assess the overall statistical significance of the factors that affect national stability (see Table 6). The results show that the regression model is statistically significant: *F*_(3, 280)_ = 20.681, *p* < 0.001. This indicates that the independent variables (national identity, gender roles, and identity and gender integration) in the model can reliably predict national stability. The variance explained by the regression (SS = 9.877) is significantly greater than the residual variance (SS = 44.577), indicating that the model has statistically significant predictive value. Overall, the ANOVA findings support the validity of the regression model and provide a basis for further testing of the research hypotheses.

**Table 6:** Analysis of variance (ANOVA) of the regression model for predicting national stability.

ANOVA						
Model	Estimate	Sum of squares	df	Mean square	*F*	*p*
M_1_	Regression	9.877	3	3.292	20.681	<0.001
	Residual	44.577	280	0.159		
	Total	54.455	283			

M_1_ includes NI_mean, GR_mean, NG_mean.

The intercept model is omitted, as no meaningful information can be shown.

Source: Compiled by the authors based on an online survey conducted in 2026.

To clearly identify the factors that influence national stability, we analyzed regression coefficients (Table 7). The results show that national identity has a statistically significant positive effect on national stability (β = 0.220, *t* = 3.439, and *p* < 0.001). In other words, as the level of national identity increases, so does the perception of national stability. Additionally, identity and gender integration showed a significant positive correlation with national stability (β = 0.254, *t* = 3.974, and *p* < 0.001), indicating that these factors are among the strongest predictors of national stability. On the other hand, the gender roles variable did not show a statistically significant effect (β = −0.075, *t* = −1.382, and *p* = 0.168), suggesting that the direct influence of gender attitudes on national stability may be weak or non-existent. Overall, these findings suggest that national identity and social cohesion are crucial to maintaining a stable society.

**Table 7:** Regression coefficients of factors affecting national stability.

Coefficients						
Model	Estimate	Unstandardized	Standard error	Standardized	*t*	*p*
M_0_	(Intercept)	3.164	0.026		121.561	<0.001
M_1_	(Intercept)	1.987	0.437		4.550	<0.001
	NI_mean	0.228	0.066	0.220	3.439	<0.001
	GR_mean	−0.166	0.120	−0.075	−1.382	0.168
	NG_mean	0.239	0.060	0.254	3.974	<0.001

Source: Compiled by the authors based on an online survey conducted in 2026.

**Figure 1:**
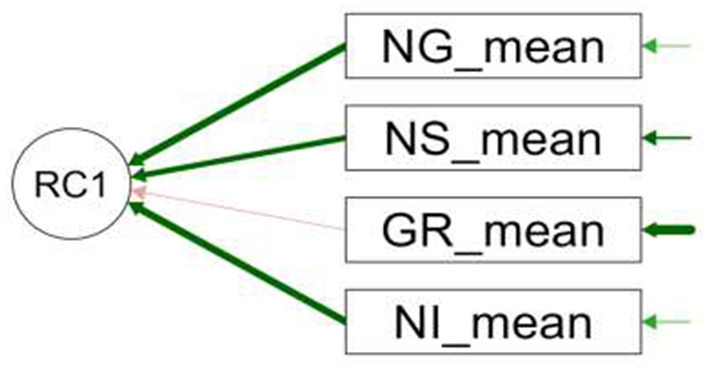
Path diagram showing the factor structure of variables. Source: compiled by the authors based on an online survey conducted in 2026.

Figure 1 shows the factor structure of the research variables. The results showed that all the main variables (national identity, national stability, identity, and gender integration) are associated with a high loading on one common latent factor (RC1).

In particular, the variables identity and gender integration (NG_mean), national identity (NI_mean), and national stability (NS_mean) showed strong positive loadings on the factor, indicating that they form a single structural system.

On the other hand, the variable gender roles (GR_mean) showed a relatively weak relationship, suggesting it is not fully compatible with the other variables and can be considered a separate construct.

Overall, the diagram visually confirms that national identity and social integration play a central role in the research model, while the influence of gender roles is limited.

**Figure 2:**
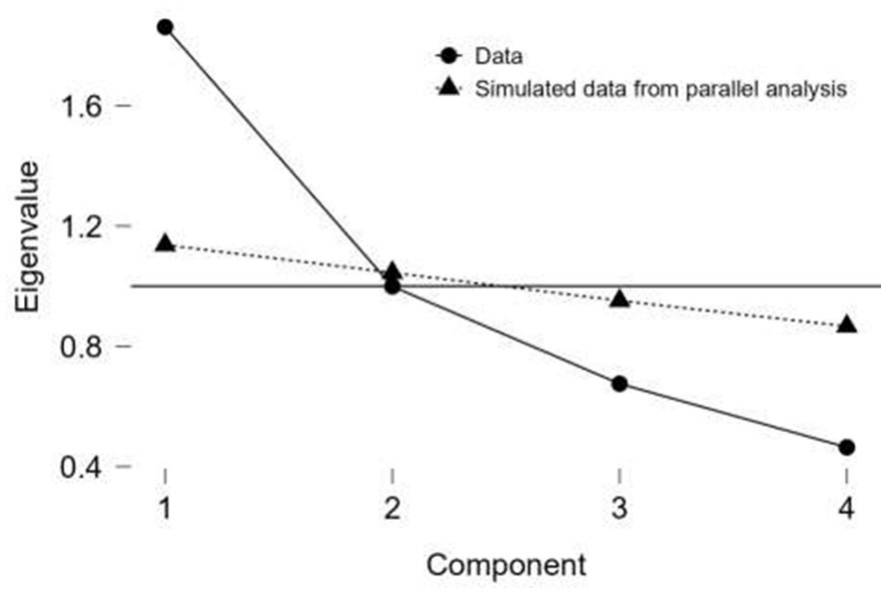
Scree plot and parallel analysis results. Source: compiled by the authors based on an online survey conducted in 2026.

Figure 2 shows the results of the scree plot and parallel analysis obtained to determine the number of factors.

The graph shows that the eigenvalue of the first component is clearly high, while the values of the second and subsequent components decrease immediately. This indicates that the “breaking point” is located after the first component.

The results of the parallel analysis also confirm this: only the eigenvalue of the first component exceeds the value obtained from random data, whereas this is not observed for the remaining components.

These results suggest that there is only one major factor in the data, and that all the main variables are related to it. The scree plot and parallel analysis generally support the unidimensionality of the factor structure and the stability of the model. In conclusion, the findings suggest that quantitative analysis statistically proves that national identity and gender integration are the decisive factors in the formation of national stability, rather than purely gender attitudes.

## 3 Discussion

This study aimed to empirically investigate the complex and multifaceted relationships between national identity, gender roles, and state stability in Kazakh society. Using multiple regression analysis and factor modeling, we gained a better understanding of the multidimensional nature of national stability formation. Our findings led us to draw several important and unexpected conceptual conclusions with implications for political science research.

While these findings are statistically significant, it is crucial to properly contextualize the explanatory scope of our model. As demonstrated in Table 5, the regression model explains 18.1% of the variance in national stability. This indicates that while the integration of national identity and gender roles is a highly meaningful predictor, the remaining 81.9% of the variance remains unexplained. We acknowledge that national stability is a highly complex phenomenon, and our model does not account for other critical macro-level predictors such as economic security, institutional trust, interethnic relations, and religiosity. Therefore, the results discussed below should be interpreted as highlighting one vital, previously overlooked dimension of stability, rather than providing an exhaustive explanation.

The main empirical novelty of the study is that the variable “Uniqueness and Gender Integration” (NG_mean) proved to be the strongest and most decisive predictor (β = 0.254, *p* < 0.001) of national stability. This indicator is even more important than the fundamental national identity itself (β = 0.220). This result proves that the institutional trust and political stability of Kazakh society cannot be ensured by either pure ethno-cultural traditionalism or global Western-style modernization, but requires an organic combination of these two contradictory phenomena.

Theoretically, this phenomenon supports the thesis of Li et al. (2025) that global and national identities are not antagonistic, but rather form new “hybrid identity models.” As an intersectional manifestation of this hybridity, actors in a transition society perceive traditional national norms and modern gender roles not as mutually exclusive, but rather as complementary aspects that reinforce national identity. Within the neo-institutional framework, the state is forced to balance its policies between civic and ethno-national discourses in order to maintain legitimacy and stability. This balancing also requires addressing the patriarchal legacy and formal modern human rights norms to ensure a harmonious and inclusive society.

These quantitative results are further corroborated by qualitative focus group data. For example, a 28-year-old city dweller (female respondent) expressed this hybrid view as follows: “*Our national values and modern equality are not enemies. I can fully preserve my Kazakh upbringing by building a career and becoming an independent professional who brings benefits to society. Only a country where women's rights are protected and opportunities are equal will be truly stable from within*.” In turn, a 45-year-old working man (respondent) expressed the following opinion about the intersection of patriarchal heritage and new values: “*Preserving tradition does not mean keeping women only at home. For the foundation of the nation to be strong, both our daughters and sons must have equal opportunities in public life. The ability to correctly combine tradition and innovation is our main strength and the guarantee of our stability*.”

This conclusion logically supports the conclusion of Sharipova et al. (2017), that the coexistence of civil and ethnic components of Kazakhstani identity forms the basis for interethnic harmony in modern Kazakhstan. That is, social harmony and stability in the country require balance not only at the interethnic or interconfessional level, but also, primarily, at the gender level. As Kudaibergenov et al. (2025) emphasize, the interrelationship between traditional values and social dynamics in contemporary society, national stability in Kazakhstan is legitimized by ensuring equal participation of women and men in public and political life while preserving the country's historical identity. The factor analysis diagram (Figure 1), which shows that national identity, integration, and stability form a common latent construct, statistically underscores the internal coherence of this ideological synthesis.

The second result of the study, unexpected for the scientific community and requiring discussion, is the lack of a direct, statistically significant effect of the individual variable “Attitudes to Gender Equality” (GR_mean) on national stability (β = −0.075, *p* = 0.168). Pearson correlation (Table 4) also clearly showed the absence of a direct relationship between gender roles and stability (NS; *r* = −0.092). These figures do not indicate the insignificance of the gender issue in society, but rather that it does not directly translate into political stability. How can this phenomenon be explained? These quantitative indicators are fully confirmed by the opinions of the focus group participants. For example, a 35-year-old working woman (respondent) said: “*Gender equality is certainly necessary, but the peace of the country and the stability of the state depend on something completely different—national unity. The rights of women and men are a social issue, and stability is a political concept. They are two separate worlds*.”

First, these data suggest that gender equality cannot be a major unifying factor or the core of a national ideology in transition states. Burkhanov (2017) has shown that national identity and stability in Kazakhstan are carefully constructed by the political elite and based on historical heritage, linguistic discourse, and spatial-territorial symbolism. When gender values are promoted outside this national context, in the form of a Western translation, they are not perceived as enhancing social stability. Rather, they are seen as a parallel phenomenon that does not affect stability.

In this regard, a 24-year-old male student explained that Western translation does not always fit the local context: “*Current gender themes often seem to be directly copied from the West, and they feel alien to our national identity. Although we support women's rights, these are mere slogans and cannot serve as the basis for our national security or as a unifying force for society*.” “*Our Kazakh roots are more important to us*.”

Secondly, this statistical isolation is explained by the hidden conflict between globalization processes and postcolonial neo-traditionalism. Syzdykova et al. (2024) note the burden of globalization on the transformation of Kazakh traditions, while Bolysbayeva et al. (2024) note the direct connection between radical societal processes and gender imbalance. Thus, although respondents generally support the change in gender roles during the survey (the variable *M* = 3.015), they do not see these egalitarian aspirations as a guarantee of state security and stability. This is also clearly reflected in the results of the parallel analysis and scree plot in Figure 2: the GR_mean variable remains as a separate dimension, outside the unified state construct (RC1).

A 48-year-old female focus group participant summed up this conflict between neo-traditionalism and egalitarian aspirations: “*Women need rights and equality, and I fully support it. But the internal stability of our country is ensured not by changing gender roles, but by preserving our national traditions and family values. Therefore, it is better not to confuse these issues*.”

In conclusion, the slightly negative but statistically insignificant GR_mean in Table 7 (β = −0.075) indicates an important political point: the internal stability of the state does not depend on the mere forcing of gender modernization, but on how organically this modernization is integrated into the national identity and harmonizes with the traditional and spiritual needs of society (Abikenov et al., 2025). The only way to ensure stability in society is to present gender policy not as a copy-paste from abroad, but as a natural evolution of national identity. This political statement was clarified by a 42-year-old civil servant (male) who participated in the focus group: “*We do not need to copy Western gender policy in Kazakhstan, it will only cause resistance from society, especially the older generation. Real equality and peace will be established only when it is based on our Kazakh mentality, family values, and develops naturally together with our national identity*.”

The empirical results of this study provide a significant new perspective on the theories of transitology, state-building, and public security. We have moved away from the traditional “civic-ethnic” dichotomy in the study of national stability and argued for the importance of integrating a macro-level gender dimension. Empirical evidence suggests that national values and egalitarian modernization are not mutually exclusive; rather, their synthesis can form a solid foundation for social cohesion. Scholars and policymakers studying the post-Soviet space should consider national identity as a complex construct intertwined with gender roles.

For Kazakhstan's ideological policy, these data provide a clear strategic signal: the state should not dismiss gender equality as a “foreign or Western phenomenon” in the process of modernizing its values. If the state's ideology is based only on conservative neo-traditionalism and patriarchal values that isolate women from public life, this can lead to decreased trust in state institutions among the younger generation in a globalized world and lead to polarization.

On the other hand, when national stability is combined with modern principles of gender equality, as evidenced by the positive effect of the NG_mean variable in the model (β = 0.254), society's internal stability and loyalty to the political system are strengthened.

Accordingly, when promoting national identity, the state's ideological apparatus should emphasize the leadership role of women in Kazakh society based on historical archetypes. This is not simply a copy of feminism, but rather the development of a unique form of egalitarianism that is understood by the people and consistent with core national values such as family solidarity, respect for elders, social responsibility, interethnic harmony, community cohesion, and cultural continuity (Syzdykova et al., 2024). This ideological need is also clearly emphasized by the opinions of focus group participants. For example, a 25-year-old city dweller (female) said about the danger of pure conservatism: “*If we are only taught the outdated concept that ‘a woman's place is at the hearth,' young people like me will lose faith in the state and its policies. We love our country, but we need equal opportunities to develop as independent individuals in our society*.” A 52-year-old representative of the intelligentsia (male) supplemented the idea of forming original equality by relying on national archetypes as follows: “*A Kazakh woman has never been without rights or silent in history*.” “*We need to promote it to young people, not copy the foreign feminism of the West. True equality and respect for women are our national values*.”

For gender policy, these results suggest a new approach to implementing reforms. The lack of a significant impact of gender roles on stability (*p* = 0.168) suggests that legislative initiatives related to gender equality in Kazakhstan should not be seen as “compliance with international standards” or “breaking patriarchal traditions.” Such rhetoric can provoke latent or open opposition from traditionalist groups.

Instead, gender reforms should be justified by focusing on national security, demographic stability, a just society, and the health of the nation. In this context, national security refers not only to military protection but also to social cohesion, institutional trust, resilience against social polarization, and long-term societal stability. Likewise, the “health of the nation” refers to demographic sustainability, family wellbeing, psychological welfare, and the overall quality of social relations within society. This should be done in conjunction with healthy elements of traditional values and religious spirituality. In the Kazakhstani context, traditional values refer to family solidarity, respect for elders, collective responsibility, preservation of cultural heritage, and social cohesion. Religious spirituality refers to ethical principles, compassion, moral self-discipline, and social responsibility derived from religious traditions that contribute to harmonious social relations.

The importance of this new communication paradigm was very accurately emphasized by a 45-year-old male head of the family who participated in the focus group: “*There is no need to forcefully impose Western standards or naked feminism on us. But beating and insulting a woman is completely alien to our Kazakh heart and religion. That's why we need strict laws protecting women's rights, not to catch up with Europe, but primarily for the strength of our families, the health of our generation, and the peace of our society*.”

The empirical analysis supported the constructivist theoretical model and confirmed all three hypotheses regarding the determinants of political stability.

First, the quantitative data supported the first hypothesis (H1), which posits a direct positive correlation between civic national identity and institutional trust. Statistical analysis showed that a high level of civic national identity is associated with increased institutional trust, indicating that this construct is a fundamental determinant of political stability.

Second, the study's results also support the second hypothesis (H2). Despite the acceptance of traditional gender roles within the context of neo-traditionalist political discourse in society, empirical models have shown that gender positions cannot directly predict national stability as an isolated independent variable. This finding confirms the multidimensional nature of the stability construct and suggests that political systems cannot maintain long-term equilibrium solely on the basis of conservative views.

Third, the data set strongly supports the statistical significance of the third hypothesis (H3). When modernization values and egalitarian gender perspectives are integrated into the construct of national identity, there is a strong, positive multiplicative effect on long-term political stability grounded in democratic principles. The results of this study show that political stability depends not only on a rigid institutional framework, but also on the complex constructivist synthesis of civic identity and modernization-egalitarian values in society. These findings open up new possibilities for studying political stability in transitional societies from a new theoretical perspective.

In addition, a set of institutional recommendations was formulated to improve state policy. First, in order to move gender policy beyond the narrow framework of social protection and legitimize it as a strategic tool for national stability, it is necessary to integrate it conceptually with programs for constructing national identity. Additionally, it is important to create new narratives in the media and cultural space that present gender egalitarianism as an integral part of Kazakh identity and normalize the image of an “independent woman leader who respects tradition.”

To ensure the success of these political and social measures, it is essential to introduce specialized modules in the education system that explain the link between national identity and gender equality. These institutional measures will help the younger generation to understand that the foundation of state stability is a fair environment, and they will become the most effective means of building a strong, systemic national resilience in the face of a global crisis of values.

In conclusion, this study demonstrates that the stability of Kazakhstan's state is not solely dependent on gender modernization, but also on its integration into national identity. To strengthen social cohesion, the state should not reject modern egalitarian values as foreign, but rather, shape them as part of the natural evolution of its historical code.

## 4 Conclusion

This study comprehensively analyses the interrelationship between national identity and gender roles in the formation of national stability in Kazakhstan. It offers new empirical findings for political science and sociology, and its main novelty is the evidence that state stability is best predicted by an organic combination of two values: “uniqueness” and “gender integration.” Quantitative and qualitative data show that gender values do not strengthen social stability when imposed outside the context of national meanings. Instead, real institutional trust and social agreement can only be established when the modernization process is perceived as an organic evolution of the national historical code.

The results obtained require a new strategic approach to balancing the state's ideological and gender policies. To achieve genuine egalitarianism, the state should promote gender equality as an integral part of the historical identity of the Kazakh people, rather than as a Western import. This will increase the younger generation's trust in state institutions and help prevent polarization between radical neo-traditionalism and feminism.

In addition, in order to ensure institutional synergy, it is important to acknowledge that gender policy and legislative reforms aimed at combating domestic violence should not only be framed within the context of “protecting women's rights,” but should also be justified through the lens of national security, family stability, and demographic sustainability. This comprehensive approach will significantly enhance the overall effectiveness of these reforms, while also reducing resistance from conservative groups.

As with any empirical study, this research has several limitations that require consideration. First, the sample size is limited, as the majority of respondents (69%) were urban residents and young adults (71%). Therefore, the results may not fully reflect the views of the wider population, especially those living in rural areas or belonging to older generations. Crucially, because urban and educated demographics consistently demonstrate more egalitarian views, the gender equality (GR_mean) scores in our sample are likely inflated compared to the broader Kazakhstani society. We explicitly acknowledge that this sampling bias directly contributes to the null finding regarding the isolated effect of gender roles on stability, and the scope of our generalizations should be interpreted within the boundaries of this sampled population.

Second, the cross-sectional nature of the study prevents us from accurately assessing the long-term changes in the causal relationships between variables. To address this, future research should increase sample size and compare across regions, age groups, and religious backgrounds.

Additionally, longitudinal studies that track changes in national identity and gender roles over time would provide a more comprehensive understanding of political stability in transitioning societies.

## Data Availability

The raw data supporting the conclusions of this article will be made available by the authors, without undue reservation.
